# Zebrafish *yap1* plays a role in differentiation of hair cells in posterior lateral line

**DOI:** 10.1038/srep04289

**Published:** 2014-03-06

**Authors:** Siau-Lin Loh, Cathleen Teh, Julius Muller, Ernesto Guccione, Wanjin Hong, Vladimir Korzh

**Affiliations:** 1 Institute of Molecular and Cell Biology, Singapore; 2 Department of Biochemistry, National University of Singapore, Singapore; 3 Department of Biological Sciences, National University of Singapore, Singapore

## Abstract

The evolutionarily conserved Hippo signaling pathway controls organ size by regulating cell proliferation and apoptosis and this process involves Yap1. The zebrafish Yap1 acts during neural differentiation, but its function is not fully understood. The detailed analysis of *yap1* expression in proliferative regions, revealed it in the otic placode that gives rise to the lateral line system affected by the morpholino-mediated knockdown of Yap1. The comparative microarray analysis of transcriptome of Yap1-deficient embryos demonstrated changes in expression of many genes, including the Wnt signaling pathway and, in particular, *prox1a* known for its role in development of mechanoreceptors in the lateral line. The knockdown of Yap1 causes a deficiency of differentiation of mechanoreceptors, and this defect can be rescued by *prox1a* mRNA. Our studies revealed a role of Yap1 in regulation of Wnt signaling pathway and its target Prox1a during differentiation of mechanosensory cells.

The Hippo signaling pathway is evolutionarily conserved from fly to human. It controls organ size by regulating cell proliferation and apoptosis^1^,^2^. Recent studies linked this pathway with regulation of cell proliferation in the CNS^3^,^4^. An important role in the Hippo pathway is performed by Yes-Associated Protein 65 (Yap), the transcription regulator containing the binding domain and transactivation domain. Yap was discovered as 65 KDa protein binding to Src homology domain (SH3) of the Yes protein^5^. Homologs of Yap have since been discovered in *Drosophila* (Yorkie)^6^, human and mouse (*YAP1*)^7^, zebrafish (*yap1*)^8^, *Xenopus*^9^. YAP functions to regulate cell proliferation, growth and survival^1^,^6^,^10^,^11^. Recently YAP was found to act as a nuclear relay of mechanical signals exerted by ECM rigidity and cell shape. This regulation requires Rho GTPase activity and tension of the actomyosin cytoskeleton, but is independent of the Hippo cascade. Within this context YAP/TAZ activity was promoted by overexpression of activated Diaphanous causing F-actin polymerization and stress fibres formation^12^,^13^. The zebrafish Yap1 has been shown to play a role during the development of the brain, eyes, and neural crest^8^.

The lateral line system is a specialized sensory organ of aquatic animals for detection of changes in hydrodynamic pressure. It consists of neuromasts arranged in a particular pattern on the body surface. Each neuromast represents a central rosette of mechanosensory hair cells surrounded by mantle cells and support cells. The mechanoreceptors are functionally and morphologically related to the mechanoreceptors of the inner ear and functionally the lateral line organ is linked to the inner ear too. The lateral line system develops in result of collective cell migration of several lateral line primordia, where early events of cell differentiation and neuromast morphogenesis are taking place^14^,^15^,^16^,^17^. Another example of collective cell migration is studied in *Drosophila* border cells during oogenesis. Here the Hippo signaling pathway was found to be involved in regulation of interaction of determinants of cell polarity resulting in polarization of the actin cytoskeleton^18^.

The developmental events regulating the specification of mechanoreceptors remain not fully understood. Several signaling pathways were implicated in this process, including, but not limited to the chemokine, Wnt, Fgf and Notch^14^,^19^,^20^,^21^,^22^,^23^. These pathways induce an expression of bHLH transcription factor Atoh1 in committed precursors each of which divides in a process regulated by Atp2b1a that gives rise to a pair of mechanoreceptors^15^,^24^. Prospero-related homeobox gene 1 (Prox1a) is a transcription factor; depending on the target gene it acts as a transcription activator or repressor. Regulation of cell cycle and cell fate specification in several types of tissue, including pancreas, lens and adrenal gland requires Prox1a^25^,^26^,^27^. In the chick spinal cord Prox1 drives neuronal precursors out of the cell cycle to initiate expression of neuronal proteins during differentiation of interneurons^28^. Importantly, Prox1a is expressed in the lateral line primordium. It is necessary for mechanoreceptors in the zebrafish lateral line to differentiate and acquire functionality^29^,^30^.

The recent discovery of the GPCR-mediated signaling acting upstream of the Hippo-Yap pathway is important within a context of the lateral line development. It was found that Gi proteins linked to SDF1-CXCR4 axis act as moderate activators of Yap, whereas G12, G13 proteins linked to SDF-1 induced migration through Cxcr4 and sphingosine-1-phosphate (S1P) signals act as strong activators of Yap^31^,^32^. Given a role of S1P as an enhancer of the CXCR4-dependent effects induced by SDF1 in other contexts^33^, the net effect of Yap activation, controlled by GPCRs at different levels, could be to promote migration of the lateral line primordium. This suggested a role of Hippo-Yap pathway in development of the lateral line system in teleosts.

With this notion in mind, we analyzed the late expression pattern of *yap1* and demonstrated that this gene is highly expressed in proliferative regions, including the otic placode that gives rise to the ear vesicle and lateral line system. The morpholino-mediated knockdown (KD) of Yap1 affects development of the lateral line system in developing zebrafish. The comparative microarray analysis of transcriptome of zebrafish embryos deficient in Yap1 demonstrated that this protein regulates expression of many genes, including *prox1a* linked to differentiation of mechanosensory cells. The knockdown of Yap1 recapitulates the Prox1a deficiency in neuromast mechanosensory cells, which could be rescued by *prox1a* mRNA. Thus, our studies revealed a novel function of Yap1 in regulation of Prox1a, which in turn regulates terminal differentiation of mechanosensory cells.

## Results

### Zebrafish *yap1* is expressed in proliferative regions during embryonic development

**Figure 1:**
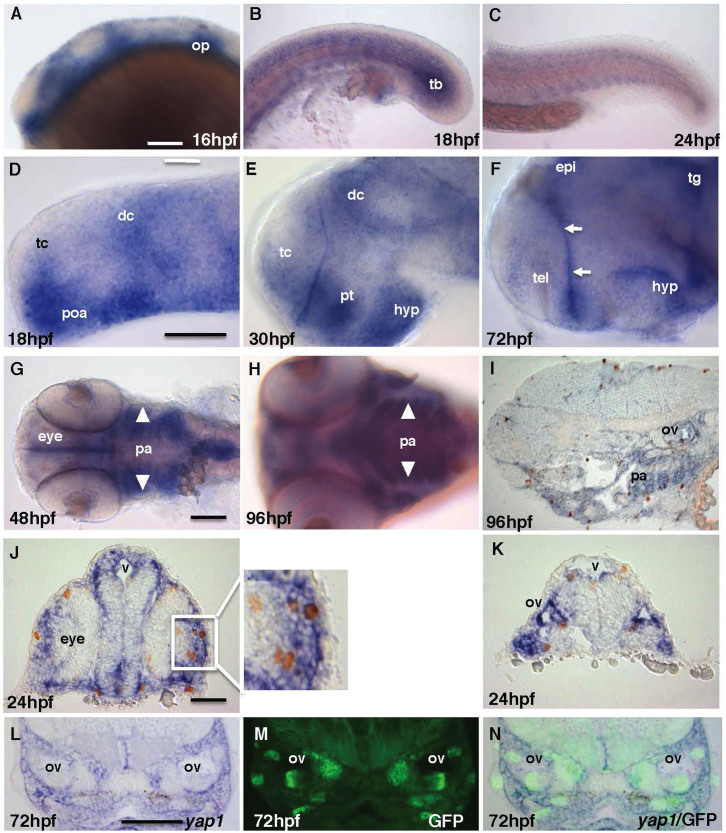
Zebrafish *yap1* is highly expressed in regions with active cell proliferation and in particular sensory progenitor cells of the inner ear. (A–C) *yap1* is expressed in the brain, somites and tail bud until mid-somitogenesis and its expression in the posterior body declines later on. (D–F) *yap1* expression become more restricted to the ventricular zones (proliferative region, arrows) in the brain as the embryo matures. (G–I) Staining of *yap1* in pharyngeal arch (arrowhead) at 48–96 hpf. (J), (K) Most proliferative cells detected by anti-pH3 antibody (brown, cell nucleus) are *yap1*-positive (blue) in cross-section of 24 hpf and (I) sagittal-section of 96 hpf embryos. L-N – cross-sections at the mid-hindbrain (ear) level of 72 hpf SqET33-mi60A transgenic larvae (lnfg, progenitors of sensory cells). (L) *yap1* in situ hybridization; (M) GFP expression; (N) composite yap1 in situ hybridization/GFP expression. (A–F) – lateral view, (G), (H) – ventral view. Scale bar = 40 μm. Abbreviations: dc, diencephalon; epi, epiphysis; hyp, hypothalamus; op, otic placode; pa, pharyngeal arches; poa, preoptic area; pt, prethalamus; tb, tail bud; tc, telencephalon; tg, tegmentum.

Previous study demonstrated that in zebrafish *yap1* transcripts are of maternal origin, but in later embryos they were detected in the notochord, brain, eyes, pharyngeal arches, and pectoral fins^8^. Here we focused on a role of Yap1 during late neural development. Therefore a distribution of *yap1* transcripts in the CNS during development up to 96 hpf was analyzed. Indeed, early on, *yap1* is expressed ubiquitously (not shown), but later its expression becomes restricted to the proliferative regions, including the ventricular zone of the neural tube (Figure 1A, D–F), pectoral fin (30 hpf onwards, not shown), and pharyngeal arches (16 hpf onwards, Figure 1G–I). Most mitotic cells with phospho-histone 3 (ph3)-positive nuclei were found within *yap1* expression domains (Figure 1I–K). It is important in the context of this paper to note that *yap1* was also expressed in the tail bud mesoderm and somites (Figure 1B, C). This analysis of the expression pattern suggests that Yap1 plays a role in cell proliferation.

### Yap1 knockdown affects the lateral line

To understand the function of Yap1 in zebrafish early neurodevelopment, we injected translational and splice-site MO against the 5′-untranslated region (5′UTR) and exon1-intron1 (E1I1) splice site at 1 to 2-cell stage and analyzed changes in Yap1 expression, general morphology of morphants, cell proliferation and apoptosis. We also studied morphant transcriptome using microarray. Both *yap1* MOs elicited similar phenotype characterized by delayed development, reduced head and eyes, cardiac edema, and curved trunk (Figure S1B, S1D). 5′UTR MO elicited more severe phenotype probably due to its effect on maternal transcripts of Yap1. The effect of E1I1 MO has been rescued by co-injection of *yap1* mRNA (Figure S1F, S2). Thus the follow-up analysis of Yap1 loss-of-function (LOF) was done in E1I1 morphants. RT-PCR of *yap1* morphant total RNA showed a reduction of the band corresponding to the *yap1* transcript and appearance of a minor band (Figure S1G). This indicated that the E1I1 MO-mediated block of a splice-site generated an abnormally spliced and unstable *yap1* mRNA that contributed to the partial LOF of Yap1. Interestingly, the gain-of-function (GOF) experiment by overexpression of *yap1* mRNA did not cause any obvious phenotype (Figure S1E, S2A, C). An injection of the anti-Yap1 MO caused extensive apoptosis, which was efficiently rescued by *yap1* mRNA (Fig. S2A). The effect of MO was transient and by 48 hpf, when the lateral line primordium (Prim1) completed its migration to the tail bud and primary neuromasts were formed, the level of cell death in the posterior lateral line was similar to that in controls (Figure S2B). At gross morphological level cell proliferation was not significantly affected in both LOF and GOF of Yap1 (Figure S2C); this observation correlates with a lack of excessive growth of embryos upon Yap1 overexpression and suggests that Yap1 acts as a permissive regulator of embryo growth during development.

To understand molecular events downstream of Yap1 we used the microarray analysis to compare gene expression in controls versus Yap1 morphants. This analysis demonstrated changes in expression of genes in the p53 and Wnt signaling (Figure S3 and not shown; Supplementary Table 1). Changes in the p53 signaling network is the likely cause of the observed apoptotic phenotype. A role of Yap1 in the Hippo pathway has been studied largely within a context of cell proliferation, but little is known about its role in regulation of genes involved in cell differentiation and specification. According to information in the Zebrafish Information Network (ZFIN) some of the genes down-regulated in Yap1 morphants were associated with detection and processing of sensory information in the eye, ear, cranial ganglia, optic tectum and ear (*ccng2*, *crx*, *fhl2b*, *icat*, *isl1*, *lnx2*, *pnp4a*, *ptpra*, *rbpms2*, *rufy3*, *tomm34*, *vsx1*). Several other genes are known to be expressed in the ear and lateral line system, including *myo1bl2*, *ndrg4*, *prox1a*, *ptena*, *sema3aa*.

### Prox1a acts downstream of Yap1

Prox1a is a target of β-catenin-TCF/LEF signaling in the mammalian hippocampus and zebrafish eye and liver^34^,^35^,^36^. It is expressed in the lateral line primordium^29^ playing a role in differentiation of mechanosensory cells^30^. Hence we decided to do more detailed analysis of its expression in *yap1* morphants. In order to avoid the non-specific effects due to apoptosis in Yap1 morphants, all follow-up experiments were performed in presence of anti-p53 MO.

**Figure 2:**
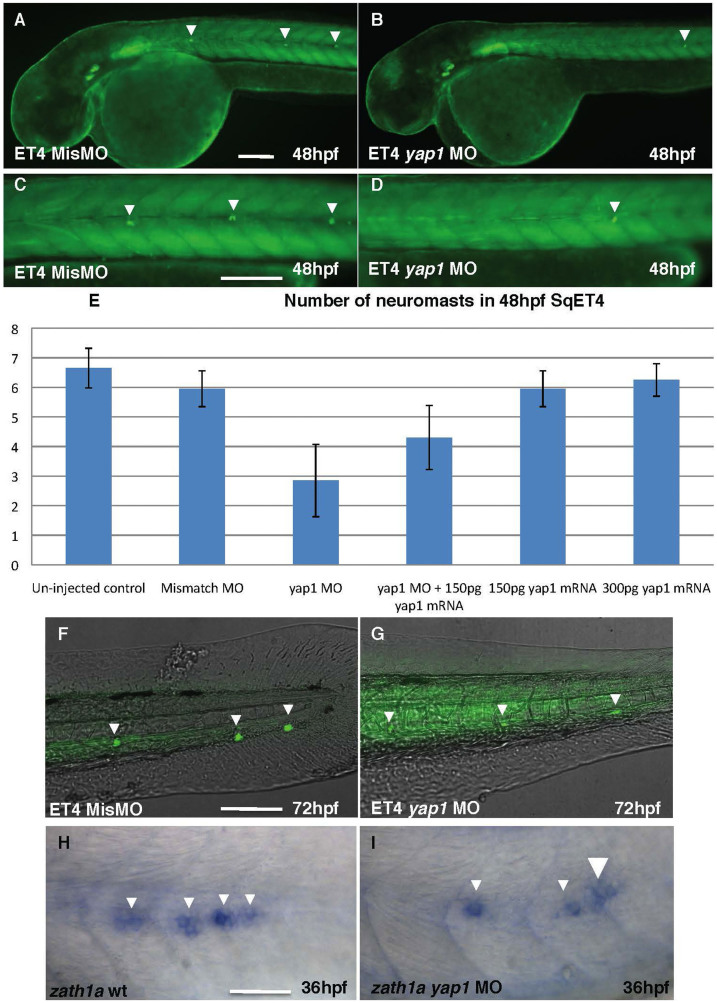
The number of neuromasts is reduced in yap1 SqET4 morphants. (A), (C), (F) – controls; (B), (D), (G) – Yap1 morphants. All embryos are shown in lateral view. The table illustrates the rescue effect of *yap1* mRNA coinjection. The original data are provided as a supplement. (A), (B) – head and anterior trunk, (C), (D) – intermediate trunk, (F), (G) – tail. Notice a reduction in a number of neuromast cells and increase of background due to longer exposure in (G) comparing to (F). (H), (I) – *zath1a* expression in the lateral line primordium of controls (H) and morphants (I). Scale bar = 40 μm, (H)&(J) = 20 μm.

SqET4 trangenics express GFP in mechanosensory cells of the lateral line^24^. MO-mediated Yap1 LOF caused a reduction in a number of neuromasts of SqET4 larvae (Figure 2B, D), in absence of such effect of the mismatch MO (Figure 2A, C). This defect was rescued in a dose-dependent manner by coinjection of MO and *yap1* mRNA. At 48 hpf both wild type control and mismatch control SqET4 embryos developed six or more neuromasts in the lateral line on each side of the trunk, whereas in Yap1 morphants only three neuromasts were detected (Figure 2E, Table 1). By 72 hpf neuromasts were detected in the tail of morphants, but GFP expression in neuromasts was significantly reduced (Figure 2F, G). This also correlated with a reduction in expression of *zath1a* in the primordium (PrimI; Figure 2H, I).

**Figure 3:**
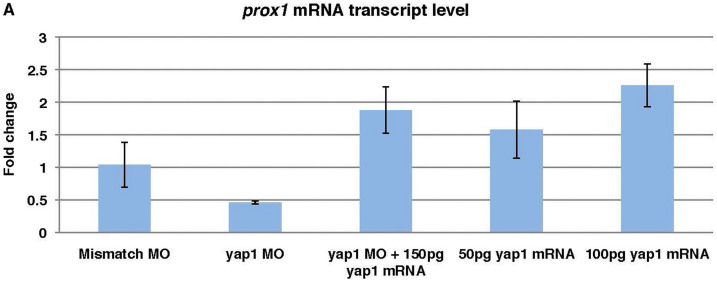
*prox1a* transcript level is *yap1*-dose-dependent. Taking un-injected control as 1 fold change, reduced *prox1a* expression level in *yap1* morphants could be rescued with *yap1* mRNA. Higher doses of *yap1* mRNA led to elevated *prox1a* expression. Mismatch control embryos showed no significant deviation from un-injected controls. Quantitative PCR results from *yap1* morphants, rescued morphants and *yap1* gain-of-function were calculated with delta CT method for fold change. The significant difference of the fold change, as compared to control with assumed fold change of one, was analyzed with two-tailed *t*-test.

Quantitative PCR showed that the transcript level of *prox1a* in the mismatch MO control is similar to that in the un-injected control, while that in *yap1* morphants was reduced below 50%. *yap1* mRNA injection into *yap1* morphants caused an increase in *prox1a* transcripts, whereas in the *yap1* GOF experiment *prox1a* transcription increased in a dose-dependent manner (Figure 3). Thus we decided to explore this phenomenon in more detail.

**Figure 4:**
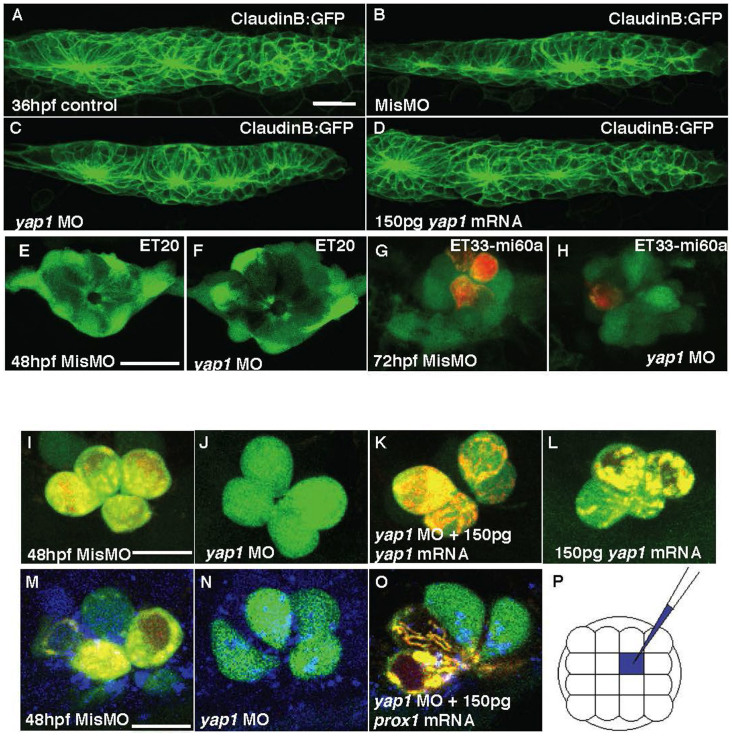
The primordium, neuromasts and mechanoreceptors of the lateral line in *yap1* morphants. (A–D) Primordium I (PrimI) in *yap1* morphants. (E) Size of PrimI at 36 hpf. (E, F) Mantle cells of neuromast in 72 hpf ET20 *yap1* morphants. (G–H) Mechanoreceptors of neuromast (red, DiAsp) in 72 hpf ET33-mi60a embryos, support cells (green). (I–L) hair cells. (J–L) Loss of *yap1* abolished the functionality of hair cells in SqET4 transgenics, stained with DiAsp (red); it was rescued with *yap1* mRNA injection. (M–O) Mismatch MO, *yap1* MO or *yap1* MO with *prox1* mRNA were co-injected with a marker (Cascade Blue) into single cell of SqET4 embryos at 16-cell stage. (P) The schematic drawing of injected single cell (blue), any one of the middle four cells, at 16-cell stage. Scale bar = 10 μm.

A number of neuromasts in *yap1* morphants was reduced even despite correction for developmental stage difference between controls and morphants. This defect in *yap1* morphants was completely rescued by *yap1* mRNA (Table 1), whereas overexpression of *yap1* did not increase a number of neuromasts (data not shown). Several reasons may cause a reduction of neuromasts, including, but not limited to, the reduction of the primordium proper, its abnormal migration, failure of neuromast deposition, and defective differentiation of hair cells. To find which of these may play a role here, *yap1* MO was injected into the claudinB:GFP transgenics, where all cells of PrimI are labeled by GFP^37^. Characterization of PrimI in morphants at 36 hpf demonstrated a reduction of size of PrimI by 28% comparing to 12% reduction in the mismatch control embryos and 9% reduction upon GOF, where the two latter values probably reflect effect of microinjection (Table 2;Figure 4A–D). Similar, the otic vesicle of morphants appears somewhat reduced, but its patterning seems to be not affected (Figure S4A–C).

To identify specific cells of neuromast affected by *yap1* LOF, the transgenic zebrafish lines with GFP expression in the mantle (SqET20), support (SqET33-mi60a), and hair cells (SqET4) were utilised^24^,^38^,^39^. Functional hair cells were also stained with the viable DiAsp dye to check whether terminal differentiation of hair cells was affected by *yap1* knockdown. This analysis showed that in morphants the support and mantle cells appeared relatively normal (Figure 4E–H), but the functional DiAsp-positive hair cells were reduced (Figure 4G–J).

In the L1 neuromast at 48 hpf (Figure 4I), 70% of hair cells were terminally differentiated and functional in mismatch control morphants similar to that in the wild type controls (61.83%, Table 3), but in the *yap1* morphants they were reduced in a dose-dependent manner upon injection of 0.05 pmol (52.5%) and 0.1 pmol morpholino (13.33%; Figure 4J, Table 3). Moreover, hair cells functionality increased in a dose-dependent manner to 22.3%, when 0.1 pmol *yap1* MO was co-injected with 75 pg of *yap1* mRNA, and to 65%, when a dose of *yap1* mRNA increased to 150 pg (Figure 4K). There was no significant difference in a number of functional hair cells (63.5%; Table 3) and neuromasts (Table 1) between controls and Yap1-overexpressing embryos (Figure 4L).

To investigate whether *prox1a* manipulation could rescue the phenotype of *yap1* morphants, *prox1a* mRNA was co-injected with *yap1* MO into 1–2 cell stage embryos. In this setup of global inhibition of Yap1 and overexpression of Prox1a, *prox1a* mRNA failed to rescue global morphology of Yap1 morphants (data not shown). This was expected since comparing to Yap1, Prox1a is expressed in more restricted manner. This suggests that Prox1a probably functions downstream of Yap1 only in some cells. To focus on interaction of these two genes in the lateral line system, *prox1a* mRNA was co-injected with *yap1* MO into one of the middle four cells fated to adopt ectoderm fate of the 16-cell stage SqET4 embryos (Figure 4M–O^24^,^40^). Cascade blue dye was added into the injection mixture as tracing dye to identify hair cells derived from injected blastomere. 77.74% hair cells in L1 neuromast were functional in mismatch morphants. In contrast, only 21.07% hair cells were functional in Yap1 morphants. Upon *prox1a* mRNA co-injection this number increased (69.6%, Table 4). This evidence strongly supported an idea that *yap1* acts not only during proliferation of mechanoreceptors in the lateral line system, but it also acts to regulate their terminal differentiation of these cells acting upstream of *prox1a*.

## Discussion

### Zebrafish *yap1* is essential for maintenance of progenitors

The fact that *yap1* is expressed in proliferative regions reaffirms its role in survival of progenitor cells in other animal models such as chick^3^,^4^. Yap1 may regulate cell proliferation differentially not only due to a high level of expression in the proliferative region. It may act later on in different proliferative regions and through multiple downstream targets many of which belong to the Wnt signalling pathway as shown by microarray analysis presented here. For example, *yap1* is expressed in pharyngeal arches from 16 hpf onwards while in somites it expression is limited by the 10–30 hpf window. Thus *yap1* probably plays an important role in regulation of cell proliferation, which is well coordinated in space and time.

The knockdown of *yap1* in zebrafish embryos reported here causes the reduction of the head, eyes and defective axis elongation in a mode similar to that observed with Yap1/yorkie LOF in *Drosophila*^6^, *Xenopus*^9^ and zebrafish^8^. This suggested that Yap1 function is conserved in evolution. The phenotype observed is also suggestive of a role of *yap1* in cell survival, as shown by an increase in cell death in *yap1* morphants. This phenotype may arise also due to reduced cell proliferation (Table 2, Figure 4C). In particular, *yap1* is essential for neural development as demonstrated by expression of this gene in regions containing neural progenitors in human fetal and adult brain^41^. Little cell death observed in the trunk of zebrafish at 48 hpf indicated that neuromast reduction was not due to an apoptosis in the lateral line system or surrounding tissue. Alternatively, it could be due to a role of Yap1 in cell proliferation during early development of the lateral line, which correlates with its expression in the posterior placodal area. The initial size of the primordium of the lateral line defines a number of neuromasts it generates^42^. Hence Yap1 expression in the proliferative cells of otic placode and a reduction of the PrimI volume in Yap1 morphants support a role of Yap1 in cell proliferation during development of the lateral line.

The two morpholino used in this study caused changes in expression of a number of genes including *prox1a*, but there were some genes which expression was affected by only one of these two morpholino. This indicates a possibility of differential splicing of *yap1*. Whereas in zebrafish so far only one *yap1* copy and only one isoform have been predicted, in other species this gene is either duplicated or expressed as a number of protein-coding isoforms, processed transcripts and mRNA containing introns. For example, in human nine *YAP1* isoforms were predicted, in mice 10 isoforms and in fugu five, one of which lacks exon 1^43^,^44^. At the same time, the number of exons of *Yap1* annotated, which often is well conserved in evolution, varies between eight (zebrafish, stickleback, tetraodon, and tilapia), nine (fugu, cod, coelacanth, medaka) and 10 (mouse and human). The mammalian gene contains two tiny exons, one of which may have been left unnoticed so far in teleosts. This warrants further study of complexity of *yap1* transcripts in zebrafish, where their specific functions could be addressed by morpholino-mediated knockdown of specific transcripts.

It has been shown that *yap1* expression is activated during cancer development^45^. Overexpression of *yap1* in embryonic zebrafish neither caused tumor formation nor led to excessive proliferation and overgrowth of organ size reported in other model animals^10^. Increase of *yap1* mRNA dose up to 750 pg per embryo only increased mortality and caused non-specific developmental defects, with LD50 at 300 pg. Similarly, an injection of mRNA of the constitutively active (Ser-87-Ala) Yap1 produced similar outcome (data not shown). While this indicates that a scenario that plays in developing zebrafish upon Yap1 GOF could be different from that in *Drosophila* development or human carcinogenesis, the nature of a transient experiment used here could be the main reason of absence of significant tissue overgrowth and/or tumor formation.

### Yap1 and mechanoreceptors

*prox1a* acts downstream of the canonical Wnt signaling^34^. Prox1a has been previously linked to terminal differentiation and functionality of hair cells in lateral line neuromasts as detected by the marker of mitochondrial activity DiAsp^30^. Since we found that *prox1a* is a downstream target of *yap1* and *yap1* knockdown phenocopies *prox1a* LOF, we investigated whether *yap1* influences hair cell functionality through *prox1a*. Similar to *prox1a* GOF, *yap1* overexpression did not cause an increase in the neuromast number and/or increased functionality of mechanoreceptors (Figure 5). In *yap1* morphants *prox1a* rescues a number of characteristics in the lateral line resulting in improved functionality of mechanoreceptors. This supported an idea that *yap1* acts in an epistatic manner upstream of *prox1a*. The cranial placodes are specialized areas of embryonic ectoderm giving rise to many sensory organs and neural ganglia of the vertebrate head. Of the seven cranial placodes, the epibranchial, otic and lateral line placodes originate from the posterior placodal area, where each placode is specified in response to additional signals^41^. *yap1* is expressed in the posterior placodal area giving rise to the lateral line and several other lineages (Figure 1). This suggests a potential functional link between *yap1* and *prox1a* in the posterior placodal area, where *prox1a* is also expressed^30^. Since *yap1* expression is absent in the primordium of the posterior lateral line and mechanosensory cell differentiation in otic vesicles was unaffected in *yap1* morphants, the regulatory interaction involving *yap1* and *prox1a* is probably indirect and may take place during development of the posterior placodal area and specification of the lateral line placode. Given the fact that Yap1 regulates a number of genes in the Wnt signaling pathway (Figure S3), an activity of this pathway well-known for its role in the lateral line development may mediate an effect of Yap1 on Prox1a^23^,^34^.

Being developed initially as a cationic mitochondrial dye DiAsp is used also to evaluate functionality of mechanosensory hair cells^46^. Since mitochondria/ATP-dependent PrimI migration is relatively normal, it does not look like that mitochondria were strongly affected by *yap1* knockdown. And yet the microarray analysis revealed a down-regulation of the gene *tomm34* encoding a translocase of the outer mitochondrial membrane subunit 34^47^. This provided a potential explanation for the deficient maturation of Prox1a-deficient mechanosensory cells evaluated by the mitochondrial dye DiAsp^30^. Since Tomm34 could be one of the most downstream components of the signaling cascade involved here, its link to Yap1-Prox1a could be studied further.

Although *yap1* is largely known as a part of Hippo pathway, which is a subject of intense studies in cell proliferation, survival, and apoptosis, the function of *yap1* in development of mechanoreceptors have not been addressed so far. Here we found and analyzed the link between *yap1* that acts in cell proliferation and *prox1a* that acts to initiate cell differentiation within a context of complex interactions taking place during regulation of terminal differentiation of mechanoreceptors. These may involve the linear pathway including Yap1-canonical Wnt-Prox1a-Tomm34. Recently, it was shown that during collective migration of border cells in *Drosophila* oogenesis Yorkie/Yap acts independently of Hippo pathway^18^. Further studies are required to clarify a role of upstream regulation of Yap1 in the lateral line development.

## Methods

### Zebrafish maintenance and manipulation

The experiments using the wild type AB strain and transgenic lines SqET4, SqET20, SqET33-mi60A^24^,^39^ and Tg(claudinB-GFP)^37^ of zebrafish (*Danio rerio*) were carried on according to the permission of the Biopolis institutional Animal Care and Use Committee (IACUC) approving the experiments (application #090430) and regulations of the IMCB Fish Facility. Pigmentation was prevented in 24 hpf and older embryos with 0.2 mM 1-phenyl-2-thiourea (PTU), and selected embryos were fixed with 4% paraformaldehyde-phosphate buffed saline (PFA-PBS) at desired development stages^48^.

### Morpholino and synthetic mRNA injection

*yap1* antisense morpholinos (MO) were designed and synthesized by Gene Tools LLC (Corvallis, USA) to target translational start site (5′UTR) and splice site (E1I1). Sequence of MOs were as follows: 5′UTR MO, 5′-ACTGAAACCGTTTTCAAGGAAAGTC-3′; E1I1 MO, 5′-GGTGGTCTCTTACTTGTCTGGAGTG-3′; 5-base-pair mismatch MO designed against E1I1 MO, 5′-GGTGCTCTGTTACTTCTCAGCAGTG-3′. The MOs were reconstituted to 1 mM with sterile water and diluted with Danieu's solution for working solution. The injected volume was 1 nL per embryo of 0.1 pmol and 0.125 pmol for mismatch/E1I1 MO and 5′UTR MO respectively at 1 to 2-cell stage. All embryos were co-injected with p53 MO at 1.5 fold of E1I1 MO or 5′UTR MO to reduce off-target neural death. Where necessary the developmental stage of morphants was adjusted to compensate for a developmental delay. *yap1* mRNA and *prox1a* mRNA were synthesized according to mMessage mMachine protocol (Ambion, USA). Embryos were injected with *yap1* mRNA encoding stabilized versions of Yap1 - T259G (S → A) and T262G (S → A). 16-cell stage injection was achieved by placing the embryo in Petri dish with moulded agar (1.5% agarose in egg water) injection wells and injecting one of the middle four blastomeres with 200 pl of working solution^24^. Cascade blue (D-1976, Invitrogen, USA) was added as tracer. To identify ectodermal derived cells that would eventually give rise to lateral line system, embryos with defined cone-shaped region of fluorescently labelled cells at 4 hpf were allowed to develop further for analysis.

### Hair cells vital staining

Mature hair cells in neuromast were labelled red by immersing live embryos in 5 mM DiAsp (4-(4-Diethylaminostyryl)-1-methylpyridinium iodide) (D3418, Sigma-Aldrich, USA) for 5 min and rinsed with egg water prior to 0.2% tricaine treatment and fluorescent imaging.

### Whole-mount *in situ* hybridization, immunohistochemistry, and cryosectioning

Probes were synthesized with either digoxigenin-labeled or fluorescine-labeled RNA antisense oligonucleotides (Roche, USA). WISH was performed according to published methods^49^. Anti-phosphohistone 3 antibodies (1:500, Millipore, USA) and anti-mouse Alexa fluor 488 stained for proliferating cells in 24 hpf whole-mount embryos. Cell death in 24 hpf and 48 hpf embryos were detected with TUNEL assay TMR red (Roche, USA). For cryosectioning, 4% PFA fixed embryos were embedded and oriented in 2% bactoagar (BDH, USA) and soaked in 30% sucrose (BD, USA) overnight. The agar blocks were trimmed and covered with Tissue-Tek O.C.T medium (Sakura, Japan) before being sectioned at 15 μm thickness using Microm HM 505 cryostat chamber (Carl Zeiss, Germany). The sections were collected with PolysineTM coated slides (Menzel GmbH and Co, Germany), and labelled for proliferating cells with anti-phosphohistone 3 antibodies and anti-mouse POD at a dilution of 1:500, before being stained with DAB (2,4-Diaminobutyric Acid) (Sigma-Aldrich, USA).

### Microarray

To identify potential Yap1 downstream targets, total DNA-free RNA was extracted from wild type embryos, *yap1* morphants (p53 MO not co-injected) and embryos injected with *yap1* mRNA at 30 hpf using RNA purification kit (Qiagen, Germany). Using the extracted RNA, a first strand of cDNA was synthesized with SuperScriptIII kit (Stratagene, USA) and oligo dT primer provided. The synthesized cDNA was further amplified and labelled with Quick Amp Labelling Kit (Agilent Technologies, USA). Putative targets down-regulated in *yap1* morphants were identified using methods in accordance to the Two-Colour Microarray-Based Gene Expression Analysis (Agilent Technologies, USA). Common down-regulated targets that occurred in both E1I1 and 5′UTR morphants that exhibited at least a two-fold decrease in expression when compared to expression of these genes in wild type embryos were selected for further analysis. The signaling pathways in the microarray data were analyzed using the DAVID bioinformatics database^50^.

### Real Time Semi-quantitative RT-PCR

Total DNA-free RNA were extracted at 30 hpf with RNA purification kit (Qiagen, *Germany*). Gene-specific primers of *actin, yap1* and *prox1a* were designed (1^st^ BASE), sequence were as follows: *actin* forward, 5′-ATGATGCCCCTGGTGCTGTTTTC-3′; *actin* reverse, 5′-TCTCTGTTGGCTTTGGGATTCA- 3′; *yap1* forward, 5′-GATAAAGCGGCCGGACACAGA-3′, *yap1* reverse 5′-AGGTGGTTTTGTTCTTGTGAT-3′; *prox1a* forward, 5′-AGAACGCGGCAACTCAAACTACA -3′; *prox1a* reverse, 5′-CCATCATGCTCTGCTCCCGAATAA-3′. Observation of band-shift in *yap1* RNA transcript of morphants was performed with One-Step RT-PCR (Qiagen, Germany) and gel electrophoresis. One-step RT-PCR with SYBR Green was performed according to manufacturer's manual (BioRad, USA) and ran in DNA Engine Opticon System (MJ Research, USA). Threshold cycle of each target gene in control and morphant was determined by using a housekeeping gene, actin, as a reference gene loading control for normalization. Fold change was calculated with delta-C(t) method and Microsoft Excel Student's two tailed *t*-test with respect to mismatch control. Primers that detect band-shift in *yap1* RNA transcript from One-step RT-PCR gel electrophoresis analysis was: 277F, 5′-GATAAAGCGGCCGGACACAGA-3′: 1027R, 5′-AGGTGGTTTTGTTCTTGTGAT-3′.

### Image acquisition and analysis

Observation and imaging of embryos were done using microscopes: Olympus AX70 (Olympus, Japan), Zen LSM700 and Zeiss Axioplan 2 (Carl Zeiss, Germany). Brightness and contrast, maximum projection of images and PrimI volume measurement were processed using ImageJ (NIH, USA) and Adobe Photoshop (Adobe Systems, USA).

## Author Contributions

Conceived and designed experiments: W.J.H., S.L.L., V.K. Performed the experiments: S.L.L., C.T., V.K. Analyzed the data: S.L.L., J.M., E.G., V.K. Wrote the paper: S.L.L., V.K.

## Supplementary Material

Supplementary Information Zebrafish yap1 plays a role in terminal differentiation and functionality of hair cells in posterior lateral line.

## Figures and Tables

**Table 1:** The number of neuromasts is reduced in *yap1* SqET4 morphants

Embryos	Number of neuromasts					
48 hpf control	Un-injected control	Mismatch MO	*yap1* MO	*yap1* MO + 150 pg yap1 mRNA	150 pg *yap1* mRNA	300 pg *yap1* mRNA
1	6	5	0	3	6	6
2	7	7	1	3	5	6
3	8	6	2	3	6	6
4	6	6	1	4	5	6
5	7	6	4	4	5	7
6	7	5	4	3	6	6
7	6	6	3	2	6	6
8	8	6	2	5	5	7
9	7	7	2	5	6	7
10	7	6	3	4	7	6
11	6	6	4	5	6	6
12	6	6	4	5	6	6
13	6	5	3	6	6	6
14	7	7	4	4	7	5
15	7	5	2	6	6	6
16	6	6	4	5	6	6
17	6	6	3	5	6	7
18	6	6	4	4	7	7
19	7	6	4	5	6	6
20	7	6	3	5	6	7
Average	6.65 ± 0.67	5.95 ± 0.60	2.85 ± 1.22	4.3 ± 1.08	5.95 ± 0.60	6.25 ± 0.55

**Table 2:** PrimI volume (μm^3^)

36 hpf embryos	Un-injected control	Mismatch MO	*yap1* MO	150 pg *yap1* mRNA overexpression
1	93951.70	85101.09	66624.06	83936.00
2	91343.15	85275.50	53287.04	96432.14
3	90352.86	83684.40	69950.38	76964.15
4	80658.73	72548.66	65498.25	78275.99
5	83206.31	85557.68	51501.79	75647.91
6	110148.70	71752.63	76168.50	76688.70
7	97783.95	72515.25	87276.48	79408.34
8	86273.50	82327.12	29689.07	81211.77
9	81884.88	76362.45	83427.23	88952.49
Average	90622.64 ± 9312.42	79458.308 ± 6060.98	64824.76 ± 17867.26	81946.39 ± 6850.83

Volume of PrimI at 36 hpf measured in μm^3^ was more significantly affected in LOF of *yap1* (n = 9).

**Table 3:** Percentage of functional hair cells in L1 neuromast (%)

48 hpf Embryos	Un-injected control	Mismatch MO	0.1 pmol *yap1* MO	0.05 pmol *yap1* MO	*yap1* MO + 75 pg *yap1* mRNA	*yap1* MO + 150 pg *yap1* mRNA	150 pg *yap1* mRNA
1	4/6 (66.67%)	4/6 (66.67%)	0/4 (0%)	3/4 (75.00%)	1/3 (33.33%)	1/2 (50.00%)	2/6 (33.33%)
2	2/4 (50.0%)	2/4 (50.0%)	1/3 (33.33%)	3/6 (50.0%)	0/2 (0%)	2/4 (50.0%)	3/5 (60.0%)
3	4/6 (66.67%)	2/4 (50.0%)	0/2 (0%)	2/4 (50.0%)	1/2 (50.0%)	2/4 (50.0%)	2/2 (100.0%)
4	1/2 (50.0%)	3/3 (100%)	1/3 (33.33%)	2/4 (50.0%)	2/5 (40.0%)	2/4 (50.0%)	2/4 (50.0%)
5	3/5 (60.0%)	2/3 (66.67%)	0/3 (0%)	3/3 (100.0%)	2/3 (66.67%)	2/3 (66.67%)	2/3 (66.67%)
6	3/4 (75.00%)	3/3 (100.0%)	0/2 (0%)	0/2 (0%)	0/2 (0%)	4/4 (100.0%)	4/4 (100.0%)
7	2/4 (50.0%)	3/3 (100.0%)	0/2 (0%)	2/4 (50.0%)	0/1 (0%)	4/6 (66.67%)	3/6 (50.0%)
8	3/6 (50.0%)	2/4 (50.0%)	1/3 (33.33%)	2/4 (50.0%)	0/1 (0%)	1/2 (50.0%)	2/4 (50.0%)
9	2/2 100	2/4 (50.0%)	1/3 (33.33%)	2/4 (50.0%)	1/3 (33.33%)	2/2 (100.0%)	3/4 (75.00%)
10	2/4 (50.0%)	2/3 (66.67%)	0/2 (0%)	2/4 (50.0%)	0/3 (0%)	2/3 (66.67%)	2/4 (50.0%)
Average %	61.83 ± 16.2	70 ± 21.9	13.33 ± 17.2	52.5 ± 24.8	22.33 ± 25.3	65 ± 18.9	63.5 ± 22.2

**Table 4:** Percentage of functional hair cells in L1 neuromast (%)

48 hpf embryos	Mismatch MO	*yap1* MO	*yap1* MO + 150 pg *prox1* mRNA
1	2/2 (100.0%)	0/2 (0%)	4/6 (66.67%)
2	2/3 (66.67%)	0/2 (0%)	5/6 (83.33%)
3	4/5 (80.0%)	2/4 (50.0%)	4/6 (66.67%)
4	3/4 (75.0%)	0/4 (0%)	3/4 (75.0%)
5	2/4 (50.0%)	0/2 (0%)	3/4 (75.0%)
6	2/4 (100.0%)	1/2 (50.0%)	2/4 (50.0%)
7	2/2 (50.0%)	1/2 (50.0%)	4/4 (100.0%)
8	4/4 (100.0%)	0/2 0(0%)	4/4 (100.0%)
9	2/4 (50.0%)	0/2 (0%)	2/4 (50.0%)
10	3/3 (100.0%)	1/2 (50.0%)	4/6 (66.67%)
11	4/4 (100.0%)	2/4 (50.0%)	2/4 (50.0%)
12	3/4 (75.0%)	1/4 (25.0%	4/6 (66.67%)
13	4/6 (66.67%)	2/4 (50.0%)	2/2 (100.0%)
14	4/6 (66.67%)	1/3 (33.33%)	4/6 (66.67%)
15	2/3 (66.67%)	0/2 (0%)	2/4 (50.0%)
16	3/4 (75.0%)	0/4 (0%)	3/6 (50.0%)
17	4/4 (100.0%)	0/4 (0%)	4/6 (66.67%)
Average %	77.75 ± 18.5	21.07 ± 23.2	69.6 ± 17.1
