# Supplementary material for: Zebrafish yap1 plays a role in differentiation of hair cells in posterior lateral line
Source: Sci Rep. 2014 Mar 6;4:4289. doi: 10.1038/srep04289 (PMC3944368; doi:10.1038/srep04289)
Supplement: Supplementary Information — Zebrafish yap1 plays a role in terminal differentiation and functionality of hair cells in posterior lateral line. [file srep04289-s1.pdf]

Supplementary information

Zebrafish *yap1* plays a role in terminal differentiation and functionality of hair cells in posterior lateral line.

Siau-Lin Loh, Cathleen Teh, Julius Muller, Ernesto Guccione, Wanjin Hong and Vladimir Korzh

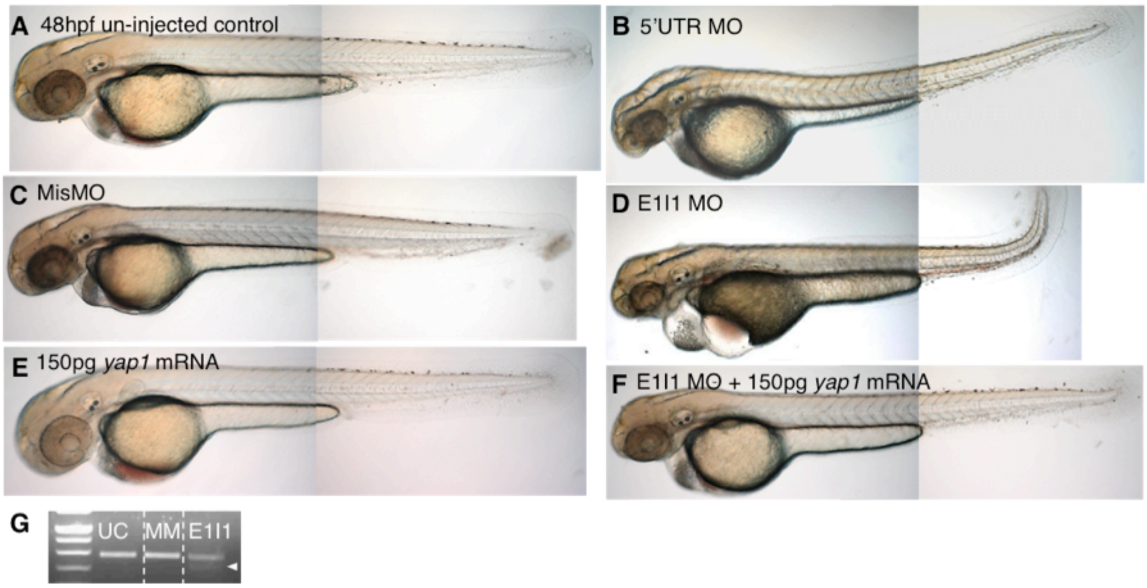

Figure S1. *yap1* morphants could be partially rescued with *yap1* mRNA.

(A) wild type controls and (C) mismatched MO, (B) Translational (5'UTR MO), and (D) splice-site (E1I1 MO) morphants, co-injected with p53 MO, have similar phenotype; curved up trunk, cardiac edema, reduced head and eye size (E) Overexpression of Yap1 caused no obvious phenotype. (F) Partial rescue was achieved by co-injection of E1I1 MO with 150pg of *yap1* mRNA. (G) Band-shift of *yap1* mRNA transcript (arrow) is observed in RT-PCR of E1I1 morphants (E1I1) as compared to un-injected control (UC) and mismatch control (MM).

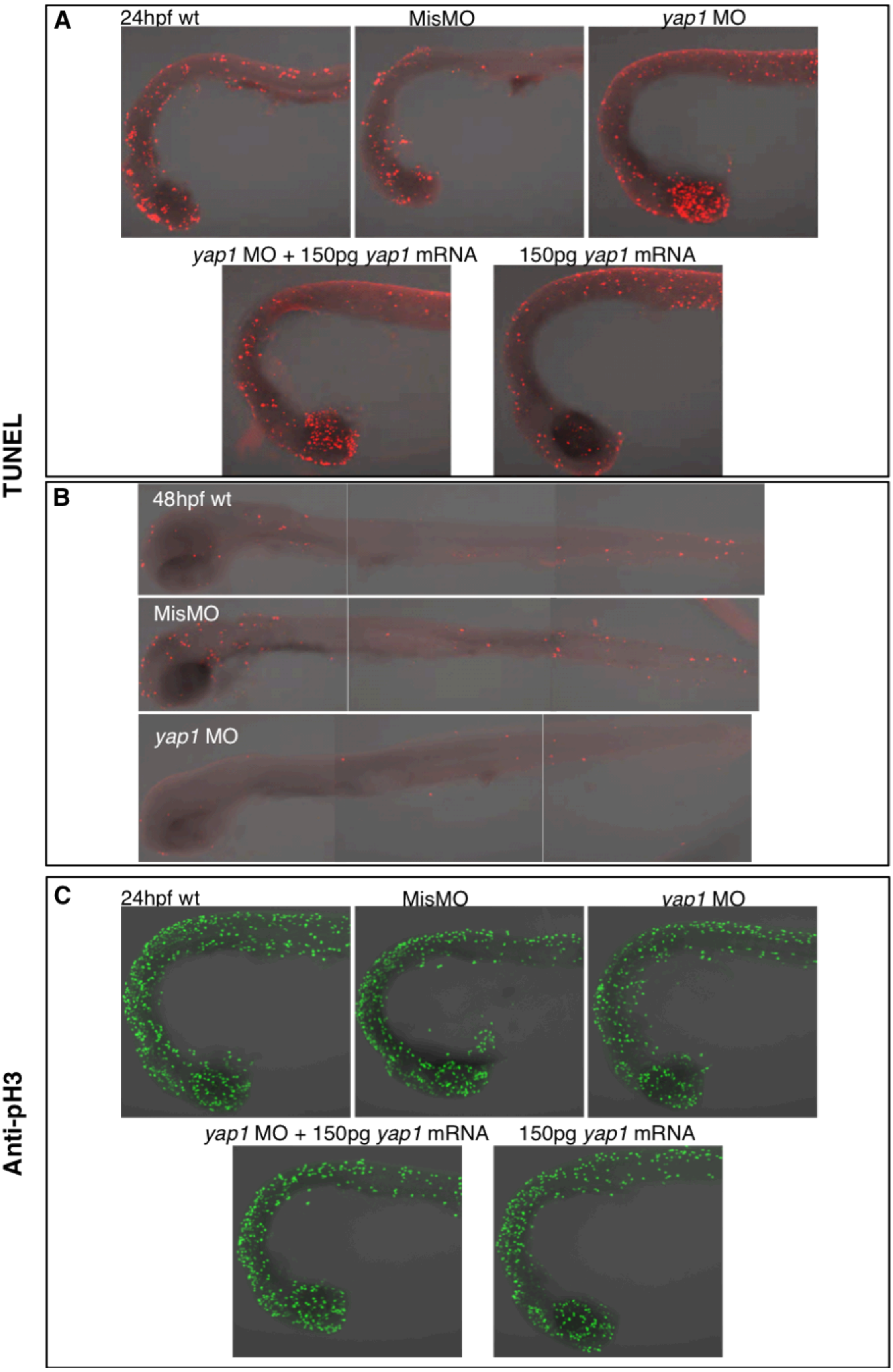
A
24hpf wt
MisMO
yap1 MO
yap1 MO + 150pg yap1 mRNA
150pg yap1 mRNA
TUNEL
B
48hpf wt
MisMO
yap1 MO
C
24hpf wt
MisMO
yap1 MO
yap1 MO + 150pg yap1 mRNA
150pg yap1 mRNA
Anti-pH3

Figure S2. Knockdown of *yap1* increased apoptosis.

(A) TUNEL-positive cells (red) were increased in *yap1* morphant mostly in the head, which could be partially rescued with co-injection of *yap1* mRNA. (B) No significant cell death was observed in posterior lateral line system of *yap1* morphants at 48hpf. (C) Cell proliferation in both gain and loss-of-function of *yap1* was not obviously different from that in controls (anti-pH3 antibody staining, green).

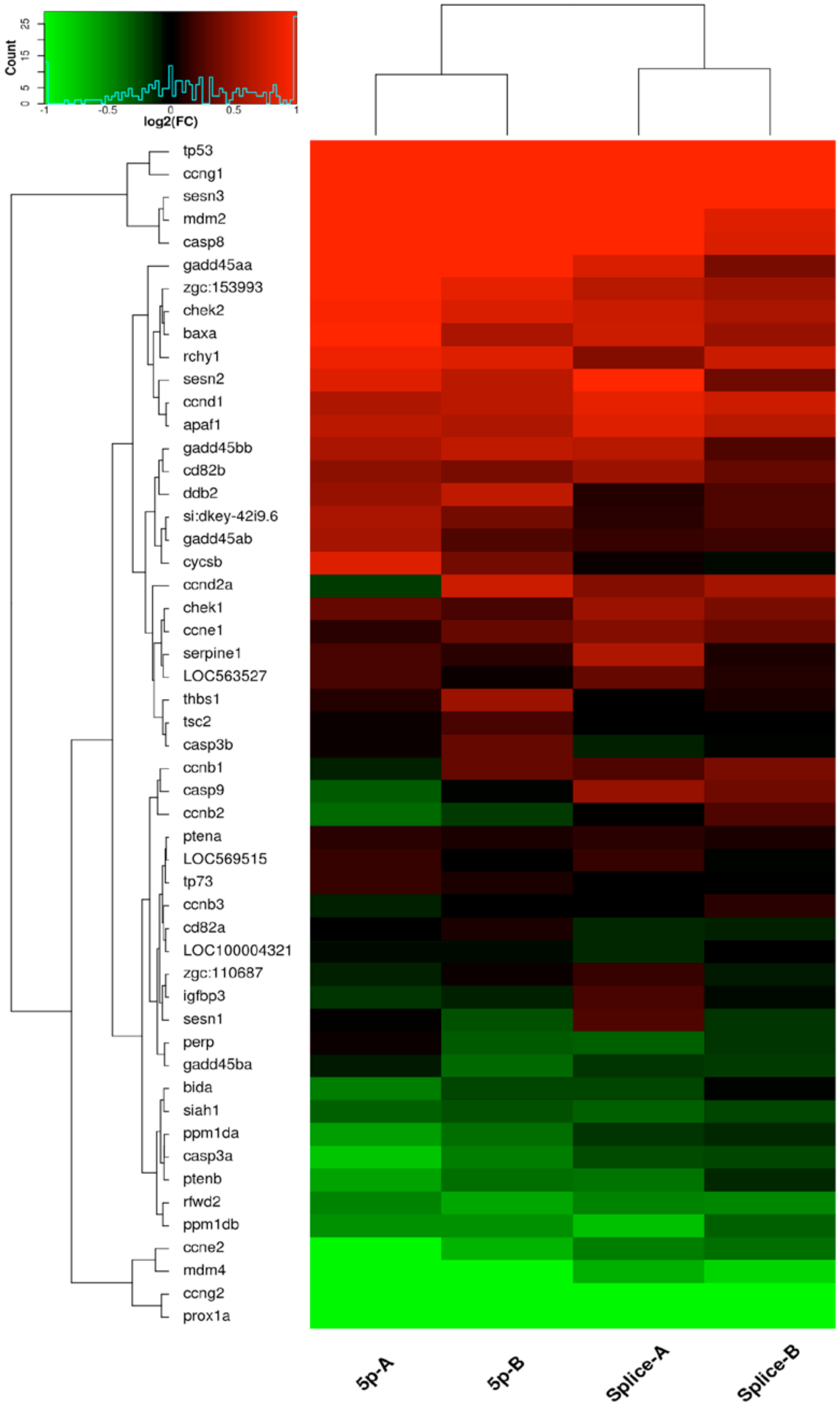
Count
0 5 15 25
-1 -0.5 0 0.5 1
log2(FC)
tp53
ccng1
sesn3
mdm2
casp8
gadd45aa
zgc:153993
chek2
baxa
rchy1
sesn2
ccnd1
apaf1
gadd45bb
cd82b
ddb2
si:dkey-42i9.6
gadd45ab
cycsb
ccnd2a
chek1
ccne1
serpine1
LOC563527
thbs1
tsc2
casp3b
ccnb1
casp9
ccnb2
ptena
LOC569515
tp73
ccnb3
cd82a
LOC100004321
zgc:110687
igfbp3
sesn1
perp
gadd45ba
bida
siah1
ppm1da
casp3a
ptenb
rfwd2
ppm1db
ccne2
mdm4
ccng2
prox1a
5p-A
5p-B
Splice-A
Splice-B

Figure S3. Morpholino-mediated LOF of Yap1 activity results in changes in expression of a number of genes, in particular in the Wnt signaling pathway.

Bioinformatics analysis of changes in expression of genes involved in the Wnt signaling is shown as a heat map. Genes up-regulated comparing to the control are shown in red and genes down-regulated in green. One of the genes with consistently reduced expression in all four LOF experiments is *prox1*.

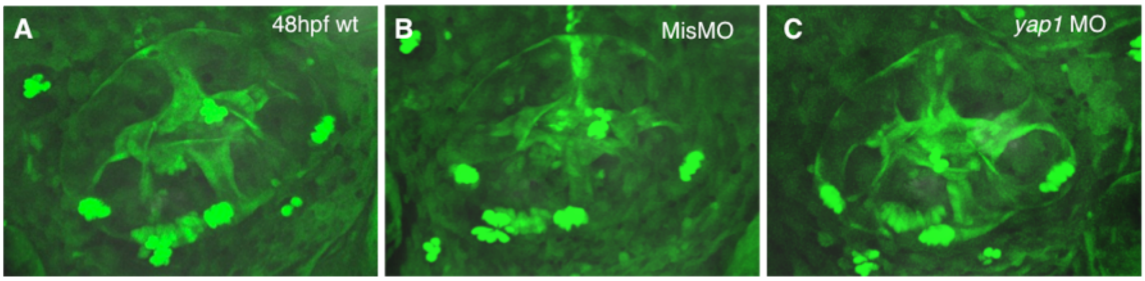

Figure S4. Otic vesicle.

The otic vesicle of morphants appears somewhat reduced, but its patterning is relatively normal.

Supplementary Table 1

Description		
Danio rerio N-myc downstream regulated gene 4 (ndrg4), mRNA [NM_001045173]	lateral line	
Danio rerio regulator of G-protein signaling 20 (rgs20), mRNA [NM_001002431]	whole organism	
Danio rerio RNA binding protein with multiple splicing 2 (rbpms2), mRNA [NM_200259]	endoderm	
AGENCOURT_85522108 NIH_ZGC_23 Danio rerio cDNA clone IMAGE:8729890 5', mRNA sequence [EE300436]	lateral line, etc	
Danio rerio translocase of outer mitochondrial membrane 34 (tomm34), nuclear gene encoding mitochondrial protein, mRNA [NM_199638]		
Danio rerio zgc:92467 (zgc:92467), mRNA [NM_001003494]		
Danio rerio hypothetical protein LOC100002393 (LOC100002393), mRNA [NM_001111239]		
Hypothetical protein (Fragment). [Source:UniProtKB/TrEMBL;Acc:Q08BF9] [ENSDART00000101146]		
Q9BQS9_HUMAN (Q9BQS9) TAFII140 protein (Fragment), partial (3%) [TC328276]		
Danio rerio visual system homeobox 1 homolog, chx10-like (vsx1), mRNA [NM_131333]		
Danio rerio pre-B-cell leukemia transcription factor 3b (pbx3b), transcript variant 2, mRNA [NM_131616]	whole organism	
Danio rerio protein tyrosine phosphatase, receptor type, A (ptpra), mRNA [NM_131888]	whole organism	
Danio rerio cyclin G2 (ccng2), mRNA [NM_213172]		
Novel protein similar to rodent myosin Ib (MYO1B) (Fragment). [Source:UniProtKB/TrEMBL;Acc:Q7T158] [ENSDART00000037459]	lateral line placode	
Danio rerio Pcdh mRNA for protocadherin2-gamma-v5-sCP1-2, complete cds. [AB160976]		
Danio rerio short stature homeobox 2 (shox2), mRNA [NM_201196]		
Danio rerio RUN and FYVE domain containing 3 (rufy3), mRNA [NM_001014335]		
Danio rerio cone-rod homeobox (crx), mRNA [NM_152940]		
Lnx2 protein (Fragment). [Source:UniProtKB/TrEMBL;Acc:Q5BJA9] [ENSDART00000046211]	lnx2b - liver, etc	
Q9PTS6_BRARE (Q9PTS6) Chico protein, partial (96%) [TC306297]		
Novel protein similar to vertebrate muscle RAS oncogene homolog (MRAS) (Fragment). [Source:UniProtKB/TrEMBL;Acc:Q1MTL0] [ENSDART00000091348]		
Danio rerio zgc:153235 (zgc:153235), mRNA [NM_001076580]		
Danio rerio zgc:110191 (zgc:110191), mRNA [NM_001020570]		
Danio rerio islet1 (isl1), mRNA [NM_130962]	liver, etc	
Danio rerio novel protein similar to mouse microtubule-associated protein 7 (Mtap7) (LOC100005195), mRNA [NM_001100092]		
Danio rerio beta-catenin-interacting protein (icat), mRNA [NM_131594]		
Danio rerio semaphorin 3aa (sema3aa), mRNA [NM_131060]		
Danio rerio zgc:86889 (zgc:86889), mRNA [NM_001002102]		
**Danio rerio prospero-related homeobox gene 1 (prox1), mRNA [NM_131405]**	**liver, lateral line, etc.**	
PREDICTED: Danio rerio similar to skin mucus antibacterial l-amino acid oxidase (LOC556554), mRNA [XM_679381]		
Danio rerio zinc finger protein, multitype 2b (zfpm2b), mRNA [NM_001039524]		
hypothetical protein LOC559358 [Source:RefSeq_peptide;Acc:NP_001038357] [ENSDART00000104393]		
Danio rerio hypothetical protein LOC100002393 (LOC100002393), mRNA [NM_001111239]		
Novel protein similar to vertebrate FK506 binding protein 10, 65 kDa (FKBP10) (Fragment). [Source:UniProtKB/TrEMBL;Acc:A3KQS9] [ENSDART00000104447]		
Danio rerio zgc:103466 (zgc:103466), mRNA [NM_001006028]	YSL, etc	
Danio rerio cone-rod homeobox (crx), mRNA [NM_152940]		
Danio rerio septin 4a (sept4a), mRNA [NM_001037379]		
Danio rerio zgc:77407 (zgc:77407), mRNA [NM_213266]		
Danio rerio PHD finger protein 10 (phf10), mRNA [NM_200655]		
Q31JI3_THICR (Q31JI3) Major facilitator superfamily MFS_1, partial (5%) [TC346714]		
XM_682606 eph-like receptor tyrosine kinase 4 {Danio rerio} (exp=-1; wgp=0; cg=0), partial (6%) [TC316034]		
AGENCOURT_17056245 NIH_ZGC_9 Danio rerio cDNA clone IMAGE:7072876 5', mRNA sequence [CK354039]		
**Danio rerio prospero-related homeobox gene 1 (prox1), mRNA [NM_131405]**	**liver, lateral line, etc.**	
AL717309 Danio rerio embryonic inner ear subtracted cDNA Danio rerio cDNA clone BN0AA024ZC03 5', mRNA sequence [AL717309]		
Danio rerio methyl-CpG binding domain protein 3b (mbd3b), mRNA [NM_212580]		
nab30g11.y2 Zebrafish lens. Unnormalized (nab) Danio rerio cDNA clone nab30g11 5', mRNA sequence [DN862208]		
AGENCOURT_61895340 NIH_ZGC_15 Danio rerio cDNA clone IMAGE:8160129 5', mRNA sequence [DV588535]		
Q306I4_BRAFL (Q306I4) Neurotrophin (Fragment), partial (7%) [TC331256]		
AGENCOURT_16388979 NIH_ZGC_7 Danio rerio cDNA clone IMAGE:7040025 5', mRNA sequence [CF998600]		
PREDICTED: Danio rerio similar to zinc finger protein 819 (LOC562992), mRNA [XM_686355]		
PREDICTED: Danio rerio similar to mCG142610 (LOC100008030), mRNA [XM_001346311]		
Danio rerio phosphatase and tensin homolog B (mutated in multiple advanced cancers 1) (ptenb), mRNA [NM_001001822]	oncogene	
Danio rerio eukaryotic translation initiation factor 5A (eif5a), mRNA [NM_213185]	whole organism	
Q4TAN3_TETNG (Q4TAN3) Chromosome 21 SCAF7282, whole genome shotgun sequence. (Fragment), partial (34%) [TC335277]		
AGENCOURT_97638567 NIH_ZGC_7 Danio rerio cDNA clone IMAGE:9003342 5', mRNA sequence [EH999607]		
PREDICTED: Danio rerio zgc:162723 (zgc:162723), mRNA [XM_685186]		
PREDICTED: Danio rerio nectin 1 (LOC557665), mRNA [XM_680762]		
Danio rerio akt interacting protein (aktip), mRNA [NM_200105]		
